# Supplementary material for: Methionine in a protein hydrophobic core drives tight interactions required for assembly of spider silk
Source: Nat Commun. 2019 Sep 26;10:4378. doi: 10.1038/s41467-019-12365-5 (PMC6763431; doi:10.1038/s41467-019-12365-5)
Supplement: Supplementary file 1 — Supplementary Information [file 41467_2019_12365_MOESM1_ESM.pdf]

Supplementary Information

Methionine in a protein hydrophobic core drives tight interactions required for assembly of spider silk

Julia C. Heiby[1,5], Benedikt Goretzki[2,3,5], Christopher M. Johnson[4], Ute A. Hellmich[2,3,*] & Hannes Neuweiler[1,*]

[1]Department of Biotechnology & Biophysics, Julius-Maximilians-University Würzburg, Am Hubland, 97074 Würzburg, Germany.

[2]Institute for Pharmacy and Biochemistry, Johannes-Gutenberg-University Mainz, Johann-Joachim Becherweg 30, 55128 Mainz, Germany;

[3]Center for Biomolecular Magnetic Resonance, Goethe-University, Max-von-Laue-Strasse 9, 60438 Frankfurt, Germany.

[4]Medical Research Council Laboratory of Molecular Biology, Francis Crick Avenue, Cambridge CB2 0QH, United Kingdom

[5]These authors contributed equally to this work.

*Corresponding authors: U.A.H.: u.hellmich@uni-mainz.de; H.N.: hannes.neuweiler@uni-wuerzburg.de

Supplementary Tables

Supplementary Table 1: Structural statistics for the NMR solution structure of L6-NTD

**Conformational restricting restraints**	
Total NOE distance restraints	1673
intraresidue $|i = j|$	448
sequential $|i - j| = 1$	463
medium-range $1 < |i - j| < 5$	347
long-range $|i - j| \geq 5$	415
Dihedral angle restraints (Talos-N)	216
No. of restraint per residue	14.4
No. of long-range restraints per residue	3.2
**Residual restraint violations[a]**	
Average no. of distance violations per structure	
0.1-0.2 Å	6.95
0.2-0.5 Å	0
>0.5 Å	1
Average no. of dihedral angle violations per structure	
1-10°	4.4
>10°	0
**Model quality (ordered residues)[a]**	
RMSD backbone atoms (Å)	2.9
RMSD heavy atoms (Å)	2.9
RMSD bond lengths (Å)	0.02
RMSD bond angles (°)	0.25
**MolProbity Ramachandran statistics[a]**	
Most favored regions	98.4
Allowed regions	1.6
Disallowed regions	0
**Global quality scores (raw/Z score)[a]**	
Verify3D	0.39
Prosall	0.84
PROCHECK (φ-ψ)	0.47
PROCHECK (all)	-0.11
MolProbity clash score	2.80
**Model contents**	
Ordered residue ranges (HetNOE > 0.6)	0-60, 63-82, 90-108, 110-129
Total no. of residues	137
BMRB accession number	27683
PDB ID code	6QJY

[a] calculated using Protein Structure Validation Software 1.5 for using ordered residues (HetNOE > 0.6). Average distance violations were calculated using the sum over r^{-6}

Supplementary Figures

Supplementary Figure 1

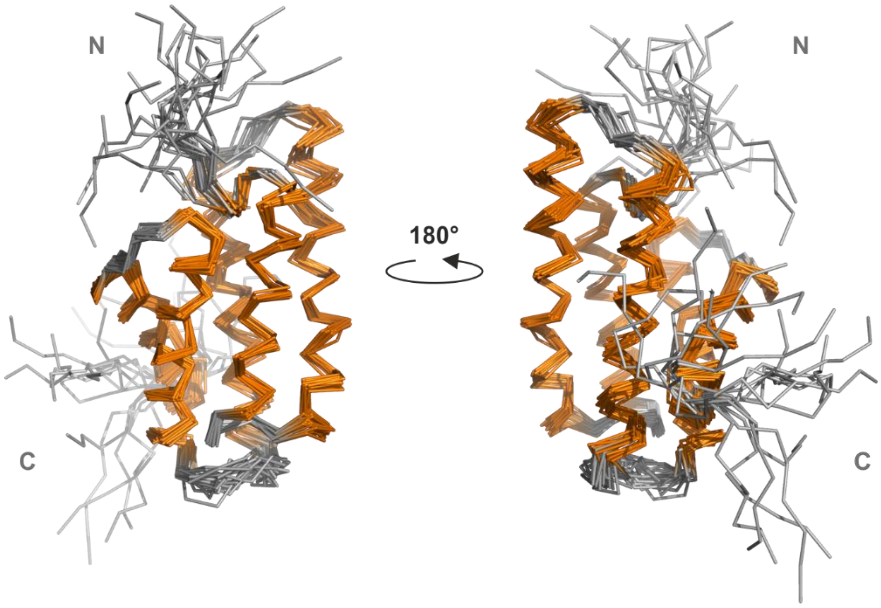

Structure bundle of L6-NTD determined by solution NMR spectroscopy. Alignment of the 20 lowest-energy conformers shown as backbone trace. The backbone heavy atom RMSD for the NMR structure bundle is 1.2 Å. Helices are highlighted orange. N- and C-terminus are indicated.

Supplementary Figure 2

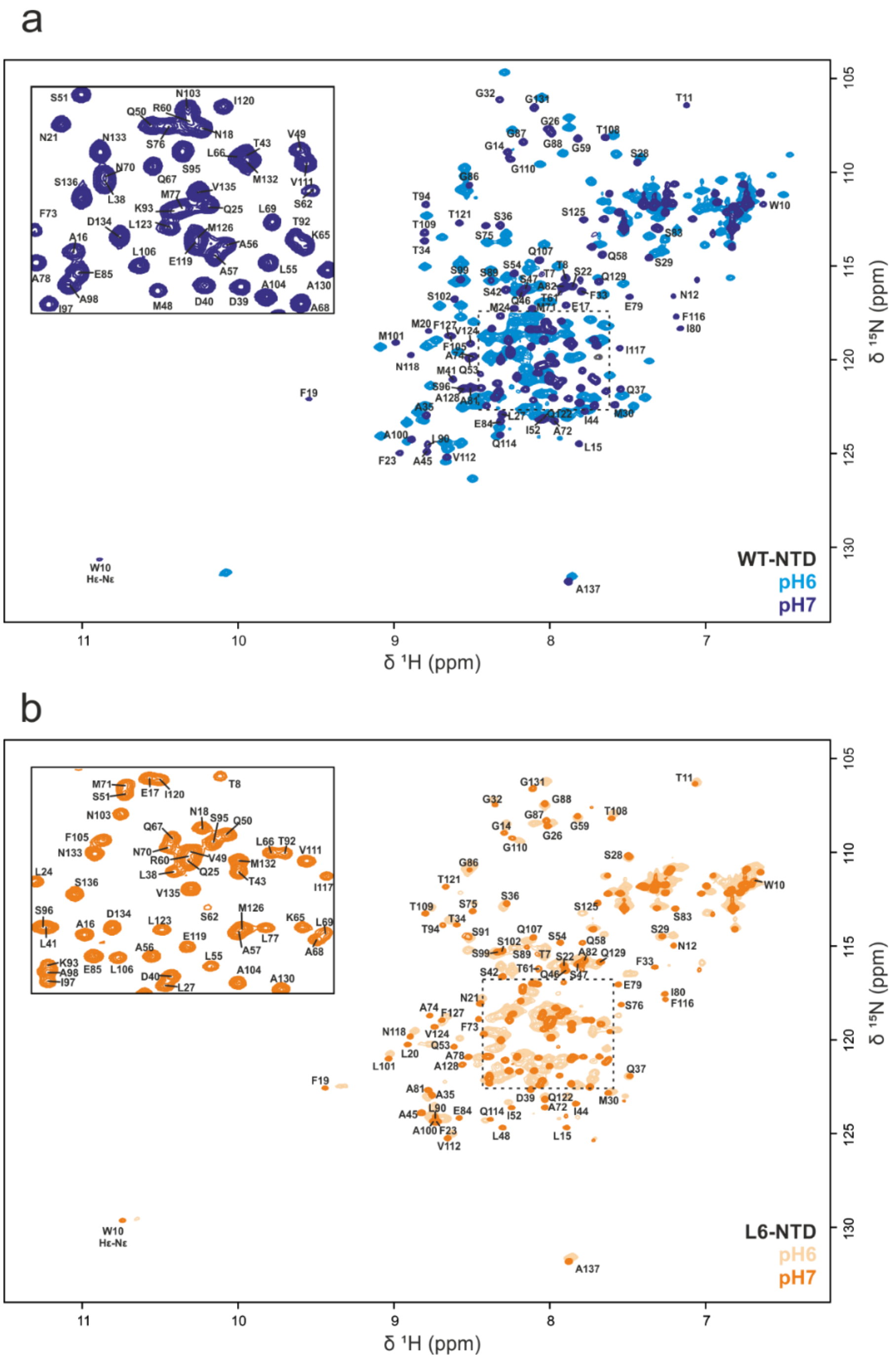

^{1}H, ^{15}N-HSQC NMR spectra of WT-NTD and L6-NTD. (**a**) Spectra of WT-NTD recorded in pH 7.0 and 200 mM ionic strength (blue) and in pH 6.0 and 8 mM ionic strength (cyan). (**b**) Spectra of L6-NTD recorded in pH 7.0 and 200 mM ionic strength (orange) and in pH 6.0 and 8 mM ionic strength (light orange). Assignments for the spectra recorded under monomer conditions (pH 7.0) are indicated (source data are provided in the BMRB; entry 18262: WT-NTD; entry 27683: L6-NTD).
